# RED: A Java-MySQL Software for Identifying and Visualizing RNA Editing Sites Using Rule-Based and Statistical Filters

**DOI:** 10.1371/journal.pone.0150465

**Published:** 2016-03-01

**Authors:** Yongmei Sun, Xing Li, Di Wu, Qi Pan, Yuefeng Ji, Hong Ren, Keyue Ding

**Affiliations:** 1 School of Information and Communication Engineering, Beijing University of Posts & Telecommunications, Beijing, P. R. China; 2 Key Laboratory of Molecular Biology for Infectious Diseases (Ministry of Education), Institute for Viral Hepatitis, Department of Infectious Diseases, The Second Affiliated Hospital, Chongqing Medical University, Chongqing, China; Roswell Park Cancer Institute, UNITED STATES

## Abstract

RNA editing is one of the post- or co-transcriptional processes that can lead to amino acid substitutions in protein sequences, alternative pre-mRNA splicing, and changes in gene expression levels. Although several methods have been suggested to identify RNA editing sites, there remains challenges to be addressed in distinguishing true RNA editing sites from its counterparts on genome and technical artifacts. In addition, there lacks a software framework to identify and visualize potential RNA editing sites. Here, we presented a software − ‘RED’ (RNA Editing sites Detector) − for the identification of RNA editing sites by integrating multiple rule-based and statistical filters. The potential RNA editing sites can be visualized at the genome and the site levels by graphical user interface (GUI). To improve performance, we used MySQL database management system (DBMS) for high-throughput data storage and query. We demonstrated the validity and utility of RED by identifying the presence and absence of C→U RNA-editing sites experimentally validated, in comparison with REDItools, a command line tool to perform high-throughput investigation of RNA editing. In an analysis of a sample data-set with 28 experimentally validated C→U RNA editing sites, RED had sensitivity and specificity of 0.64 and 0.5. In comparison, REDItools had a better sensitivity (0.75) but similar specificity (0.5). RED is an easy-to-use, platform-independent Java-based software, and can be applied to RNA-seq data without or with DNA sequencing data. The package is freely available under the GPLv3 license at http://github.com/REDetector/RED or https://sourceforge.net/projects/redetector.

## Introduction

RNA editing is one of the post- or co-transcriptional processes with modification of RNA nucleotides from their genome-encoded sequence [1]. In humans, the major types of RNA editing are adenosines to inosines (A→I editing) and cytidine to uracil (C→U), mediated by ADAR enzymes and APOBEC1 cytidine deaminase. Since I and U are interpreted as guanosine (G) and thymine (T) during splicing and translation, these changes in protein-coding sequences may lead to codon changes, and thus alter functional properties of the proteins [2]. In addition, RNA editing in the introns can affect alternative splicing, and hyper-editing of untranslated regions (UTRs) can lead to retention of mRNA inside the nucleus.

RNA editing has been linked to a wide range of human diseases, including cancer, neurological disorders, metabolic diseases, viral infection, and autoimmune disorder [3]. Paz-Yaacov et al. [4] suggested that A→I RNA editing may serve as an additional epigenetic mechanism relevant to cancer development and progression. In a recent report, Chen et al. [5] found that an A→I RNA editing of *AZIN1*, leading to a non-synonymous substitution (ser367gly) of *AZIN1*, is increased in hepatocellular carcinoma specimens. It was noted that the frequency of *AZIN1* RNA editing increases during progression from cirrhosis and primary liver cancer to advanced hepatocellular carcinoma with recurrence and metastasis [5]. The edited form of *AZIN1* has a stronger affinity to antizyme, and the resultant higher *AZIN1* stability promotes cell proliferation. Sharma et al. [6] identified transcripts of hundreds of genes undergoing site-specific C→U RNA editing in monocytes in response to hypoxia and interferons.

The events of RNA editing can be detected by target-specific RNA sequencing, comparison of genomic DNA with RNA [7], and transcriptome sequencing (RNA-seq) [8, 9]. With the advent of next-generation sequencing, a comprehensive set of several hundred human RNA editing sites has been detected by comparing genomic DNA with RNA from seven tissues of a single individual [10]. Using RNA-seq data alone from multiple samples, RNA editing sites can be called with high confidence [11]. However, there still remains challenges in identifying RNA editing sites at the genome scale in that true RNA editing sites need to be discriminated from its counterparts on genome, as well as technical artifacts (e.g., sequencing or read-mapping errors) [12, 13].

Several tools have been designed to detect high throughput RNA editing sites. Picardi and Pesole [14] provided a suite of python scripts in ‘REDItools’ to investigate RNA editing using next-generation sequencing data. REDItools was a command line tool and included several features: it requires input in binary sequence alignment/map (BAM) format, detects RNA editing candidates by comparing pre-aligned RNA-seq and DNA sequencing reads, explores the RNA editing potential of RNA-seq experiments by looking at known events, and performs the *de novo* detection of RNA editing candidates using only RNA-seq data. Distefano et al. [15] presented a web-based tool of ‘VIRGO’ that maps A→G mismatches between genomic and expression sequence tag sequences as candidate A→I editing sites. The rddChecker (http://ccb.jhu.edu/software/rddChecker/) program is a Perl tool for predicting RNA-DNA differences that might be caused by post-translation editing of the RNA. Short read alignments were analyzed to detect variable sites and then a variety of filters were employed to remove potential sequencing and alignment artifacts, as well as known SNPs. However, the program depends on both RNA-seq and DNA sequencing data, therefore, potential RNA-editing sites cannot be identified in RNA-seq alone. RCARE [16] is a web-based tool for searching, annotating, and visualizing RNA-DNA difference sites based on current knowledgebase on RNA-editing, and thereby provides evidence for improving the reliability of identified RDD sites. However, the RCARE cannot be used to identify potentially novel RNA-editing sites. Recently, Zhang and Xiao [17] presented a rigorous method (GIREMI) by calculating the mutual information of the mismatch pairs identified in the RNA-seq reads to distinguish RNA editing sites and SNPs, and a generalized linear model (GLM) was trained to achieve enhanced predictive power for identifying RNA-editing sites using RNA-seq data alone. However, a computational software (or tool) for the identification and visualization of RNA editing sites has yet to be released.

Here, we present a Java-MySQL software −‘RED’− to detect and visualize potential RNA editing sites. Using RNA-seq data alone (i.e., *de novo* mode), or using both RNA-Seq and DNA sequencing data (i.e., *DNA-RNA* mode), RED can identify potential RNA editing sites by integrating multiple rule-based and statistical filters. All potential RNA editing sites were shown at the genome levels, and a given site was visualized in its sequence context. We demonstrated the validity and utility of RED by analyzing two data sets: 1) C→U RNA editing sites in Sharma et al. [6], and 2) a RNA-seq data and whole-exome sequencing data in the liver normal tissue from a patient of hepatocellular carcinoma. The application is suitable to next-generation sequencing data related to the identification and visualization of RNA editing sites.

## Design and Implementation

**Fig 1:**
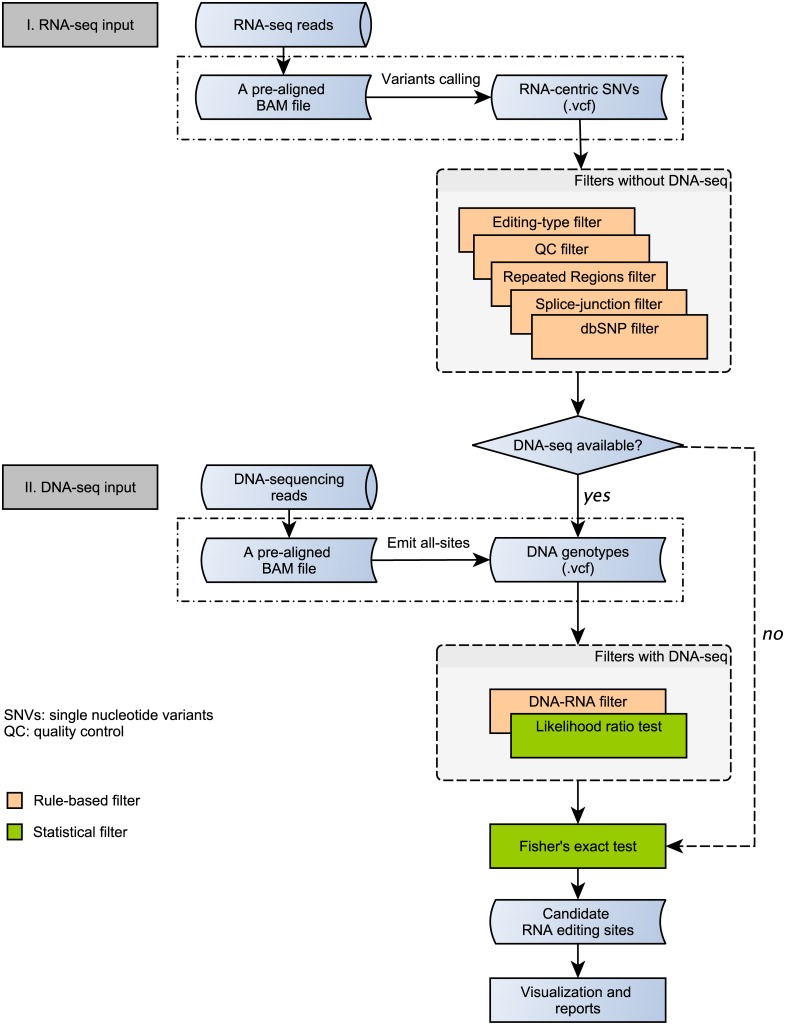
A computational framework to design RED. If DNA-sequencing data is available, potential RNA-editing sites can be identified by DNA-RNA mode; otherwise by *de novo* mode. Rule- and statistical-based filters were integrated in the framework.

The framework for the design and development of ‘RED’ was shown in Fig 1. We incorporated multiple rule-based and statistical filters [13] to remove spurious RNA editing sites, and provided a graphical user interface (GUI) to visualize RNA editing sites at the genome and the site levels. RNA editing sites can be detected using the *de novo* mode or the *DNA-RNA* mode if DNA sequencing data is available. RED can also be used in a non-GUI/command line mode.

The input required for RED are in pre-aligned BAM file and variant calling format (VCF) file. For example, the recommended workflow in GATK for RNA-central variants calling (https://www.broadinstitute.org/gatk/guide/article?id=3891) includes pre-processing and variant discovery. The pre-processing of RNA-seq reads for generating a recalibrated bam file includes mapping to reference, marking duplicates, splitting ‘N’ Trim, indel realignment, and base recalibration. Then, analysis-ready RNA-seq reads were processed to generate a VCF file by the ‘HaplotypeCaller’ walker using RNAseq mode and then raw variants were filtered in RNAseq-specific settings. If DNA sequencing data is available, genotypes could be emitted for all sites using the ‘UnifiedGenotyper’ in GATK [18, 19]. At the latest release of RED, the input can be multiple bam files and VCF file with multiple samples calling.

In RED, the BAM file was used for the visualization purpose, and the VCF file including variants information was used for detecting potential RNA-editing sites by the core algorithm. However, it should be noted that RED can be performed without BAM file.

In addition, RED requires several files to be loaded for filtering purpose, including a repeat region masked file by RepeatMasker (hg19.fa.out, http://www.repeatmasker.org), a gene annotation file in gtf format (genes.gtf, http://genome.ucsc.edu), a VCF file containing all known SNPs (dbsnp_138.hg19.vcf, http://www.ncbi.nlm.nih.gov/SNP), and a file containing known RNA editing sites (from DARNED [20] and from RADAR [21]).

### Rule-based filters

We used multiple rule-based filters to remove spurious sites caused by errors in construction of RNA-seq library and sequencing, incorrect sequence reads mapping, and germline variants in the genome. Users can view and adjust the specific filter settings when applying filters (Table 1).

**Table 1:** Measures used for filtering spurious RNA editing sites.

Filters	Sites excluded
QC filter	Sites were 1) Q < 20; or 2) depth of coverage (DP< 6)
Repeat region filter	Sites were in repeat regions (except for *SINE/Alu*)
Splice-junction filter	Sites were within ±2 bp of the splice junction
Known SNP filter	Sites were known SNPs
DNA-RNA filter	Sites whose genomic counterparts was not reference homozygote (i.e., AA)
Fisher’s exact test and FDR	FDR (*q* value) > 0.05

FDR: false discovery rate

1. RNA editing type filter: the type of RNA editing can be selected at user’s preference. In this paper, we focused on two major types: 1) A→G change which is mediated by ADAR enzymes; and 2) C→U change (e.g., mediated by APOBEC1 cytidine deaminase).
2. Quality control (QC) filter: two measures of base quality (Q) and depth of coverage (DP) were used in the QC filter. For example, a given site would be removed if it was of a low quality (e.g., Q< 20) or with a low depth of coverage (e.g., DP< 6).
3. Repeat regions filter: variants that were within repeat regions were excluded. However, sites in *SINE/Alu* regions were remained since A→I RNA editing is pervasive in *Alu* repeats [13, 22] and it has been implicated in human diseases such as breast cancer and Ewing’s sarcoma [23].
4. Splice-junction filter: variants that were within ±k bp (e.g., *k* = 2) of the splice junction, which were supposed to be unreliable [24], were excluded based on the gene annotation file.
5. Known SNP filter: RNA-seq variants that were known SNPs at DNA level were excluded for eliminating germline variants based on the VCF file containing all known SNPs.
6. DNA-RNA filter: RNA-seq variants where its counterparts in genomic DNA is not reference homozygote (e.g., AA) would be excluded if DNA sequencing data is available.

### Statistical filters

To reduce the errors in detecting RNA editing sites caused by technical artifacts (e.g., sequencing errors), we incorporated two statistical filters in RED: likelihood ratio (*LLR*) test [25] and Fisher’s exact test. We used the A→G change for illustration purpose, and it can be used in other types of RNA editing.

First, we integrated a likelihood ratio (*LLR*) test for detecting RNA editing sites [25]. For a potential RNA-editing site, we denoted *n*(*A*) as ‘A’ nucleotides, and *n*(*G*) as ‘G’ nucleotides of observed data. Likelihood ratio (*LLR*) test [25] is a probabilistic test incorporating error probability of bases (i.e., sequencing errors) for detecting RNA editing sites if DNA sequencing data is available. The likelihood of observing *n*(*A*) and *n*(*G*) at a candidate RNA editing sites in the observed sequence data *D* would be given by the binomial probability of *P*(*D*|*f*) = *f*^*n*(*A*)^(1 − *f*)^*n*(*G*)^, where *f* is the unedited fraction of RNA species. The maximum likelihood estimate of *f* is given by *f*_*ML*_ = *n*(*A*)/(*n*(*A*) + *n*(*G*)). if *max*_*f*_ *P*(*D*|*f*) is much greater than *P*(*D*|*f* = 1), i.e., the likelihood of the observed data (*D*) without RNA editing, we have a strong evidence for an RNA editing event [10]. We need to take into consideration of the probability of sequencing error in estimating *P*(*D*|*f* = 1), which can be computed using Phred base error probabilities in DNA sequencing reads. The log likelihood ratio (*LLR*) was defined as: *LLR* = *log*_10_[*max*_*f*_ *P*(*D*|*f*)/*P*(*D*|*f* = 1)]. Variation sites with *LLR* < *m* were excluded, where *m* is self-defined and *m* = 4 is suggested. The *LLR* ≥ 4 indicated that the probability of editing event happened is 10^4^ times more than that of non-editing in reality.

In addition, we assessed the significance for a given RNA editing site by comparing its expected editing levels. The expected number of *n*(*A*) and *n*(*G*) for the given site was calculated based on the known RNA editing sites from the DARNED [20] and RADAR database [21]. These numbers (expected/observed) were then used through the Fisher’s exact test to calculate the p-value of the given RNA editing sites. For correction for multiple testing, all p-values were adjusted by false discovery rate (FDR) using the method of Benjamini & Hochberg [26].

### Visualization with GUI

**Fig 2:**
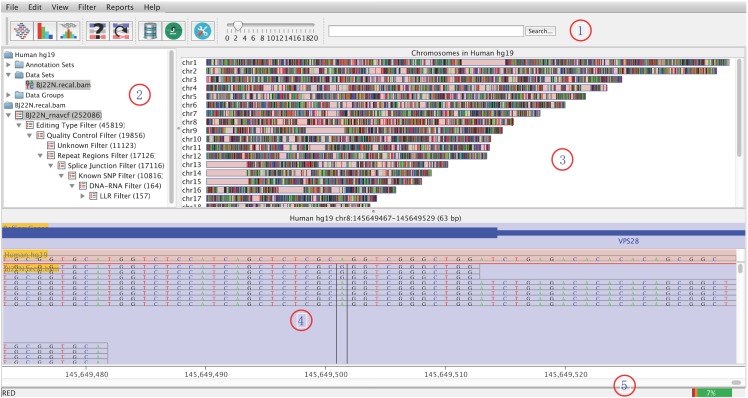
Graphic User Interface of RED. Panel #1, toolbar panel; panel #2, directory panel; panel #3, genome panel; panel #4, chromosome panel; and panel #5, status panel.

RED is designed to visualize and explore potential RNA editing sites with GUI at the genome and the site levels (Fig 2). The distribution of all potential RNA editing sites can be shown in a karyogram overview. With the idea implemented in the vigorous tool of Integrative Genomics Viewer (IGV) [27], a potential RNA editing site is highlighted in the regions of RNA-seq reads with annotation. If DNA sequencing data is available, it could be compared with its counterpart on the genome. The main window of RED consists of five panels:

1. Toolbar panel: provides a convenient way to access commonly used functions, which can be accessed via the main menu or keyboard shortcuts.
2. Directory panel: provides a quick overview of accessed objects, including annotation set, data set, site set and site lists. A site list includes the remaining variants after each step of filtering, which is shown in a hierarchical tree structure.
3. Genome panel: provides an overview of human chromosomes (i.e., chr[1-22, X, Y, M]). After RNA editing filters were applying, potential RNA editing sites would be shown in a karyogram overview.
4. Chromosome panel: contains tracks of annotation and data. The annotation track shows gene features from NCBI RefSeq, and reference sequence. The data track presents all mapped RNA-seq reads from the BAM file with two different types: genomic interval and base status.
5. Status panel: the left shows related information for a chromosome, a site or a feature when the mouse is over it, and the right is the usage of memory.

### Implementation based on Java and MySQL

RED was implemented with several key technologies, including: 1) fast data storage/retrieve and low memory usage with high-throughput next-generation sequencing data and variants data; 2) diverse filters to detect potential RNA editing sites with an improved performance; 3) information synchronization among the genome panel, annotation and data track; and 4) abundant presentation for data analysis and output.

RED is mainly developed using Java programming language, together with MySQL for data management and *R* (http://www.r-project.org) for statistical analysis. All functions related to MySQL and R were automatically executed in RED. RED can run on mainstream computational platforms, including Windows, Linux and Mac OS X.

To make the computation more efficient, everlasting and faster-saving, a widely used relational database management system (RDBMS) − MySQL − was employed to manipulate variants data (i.e., RNA-centric VCF file and DNA VCF file if available), as well as files required for filtering. RED can speed up filtration by powerful database engine and make a quick query of the filtering result (i.e., a site list). The Java Database Connectivity (JDBC) was used in RED to connect a local or remote database.

RED also used functions in *R*, a language and environment for statistical computing, to perform statistical analysis, including FDR. A simple Java library (‘rCaller’) was used to call *R* commands and scripts from Java.

RED provides flexible exports for image and text output. Images generated from RED can be exported in the format of SVG or PNG. The distribution of RNA editing sites according to its editing type and chromosome were present in graph. Information regarding to all potential RNA editing sites can be exported to a tab-delimited text file, including chromosome, position, class ID, reference base, alternative base, quality, editing level, p-value and FDR. In addition, a RED project file, including potential RNA editing site lists, information of data set and annotation set, and software preference, can be saved at user’s preference.

## Results

Sharma et al. [6] identified and experimentally validated C→U RNA editing sites in hypoxic but not normoxic monocyte-enriched peripheral blood mononuclear cells (MEPs, three samples per group). We tested the validation of RED and REDItools (version 1.0.3) using these validated C→U RNA editing sites. RNA Sequencing data of six samples were downloaded from NCBI Sequence Read Archive with accession number SRP040806. We followed the GATK Best Practices workflow for calling variants in RNA-seq (https://www.broadinstitute.org/gatk/guide/article?id=3891) with default settings. A Linux server of 256 GB memories and 4 CPU (40 core, Intel(R) Xeon(R) CPU E7- 8850 @ 2.00GHz) was used for the computation. A detail of the software and commands used in the analysis is present in the S1 Text.

**Fig 3:**
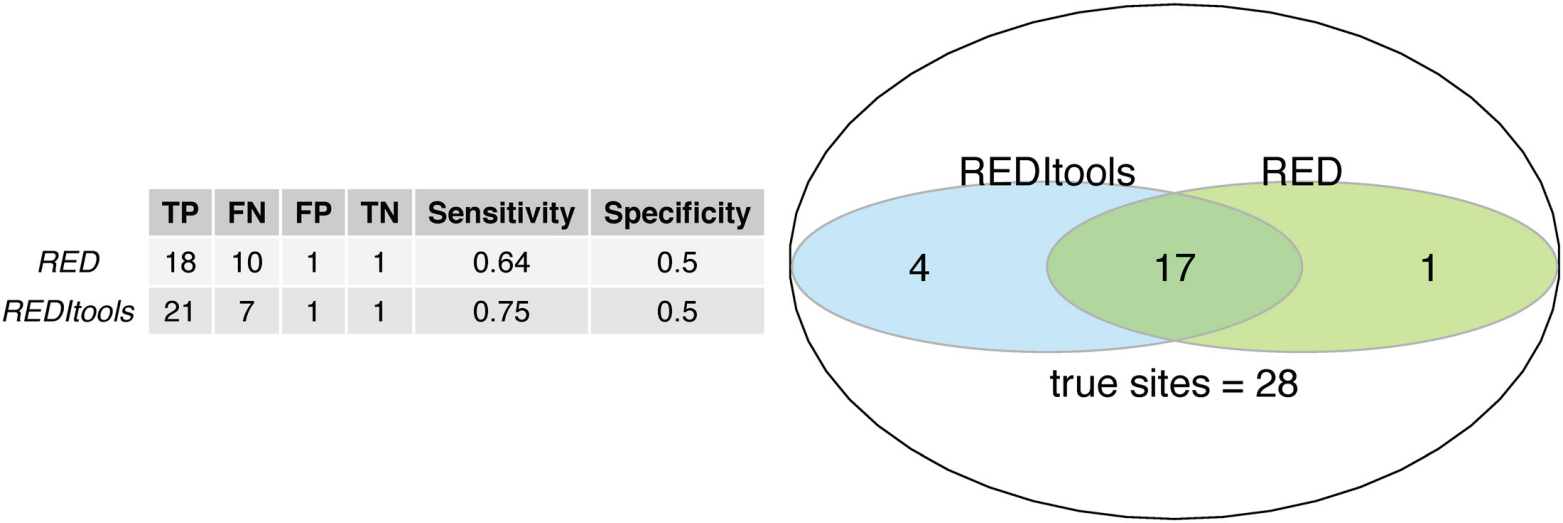
A comparison of validation for identifying RNA-editing sties using RED and REDItools in an analysis of a sample data-set with 28 experimentally validated C→U RNA editing sites. TP, true positive; FP, false positive; TN, true negative; and FN, false negative.

**Fig 4:**
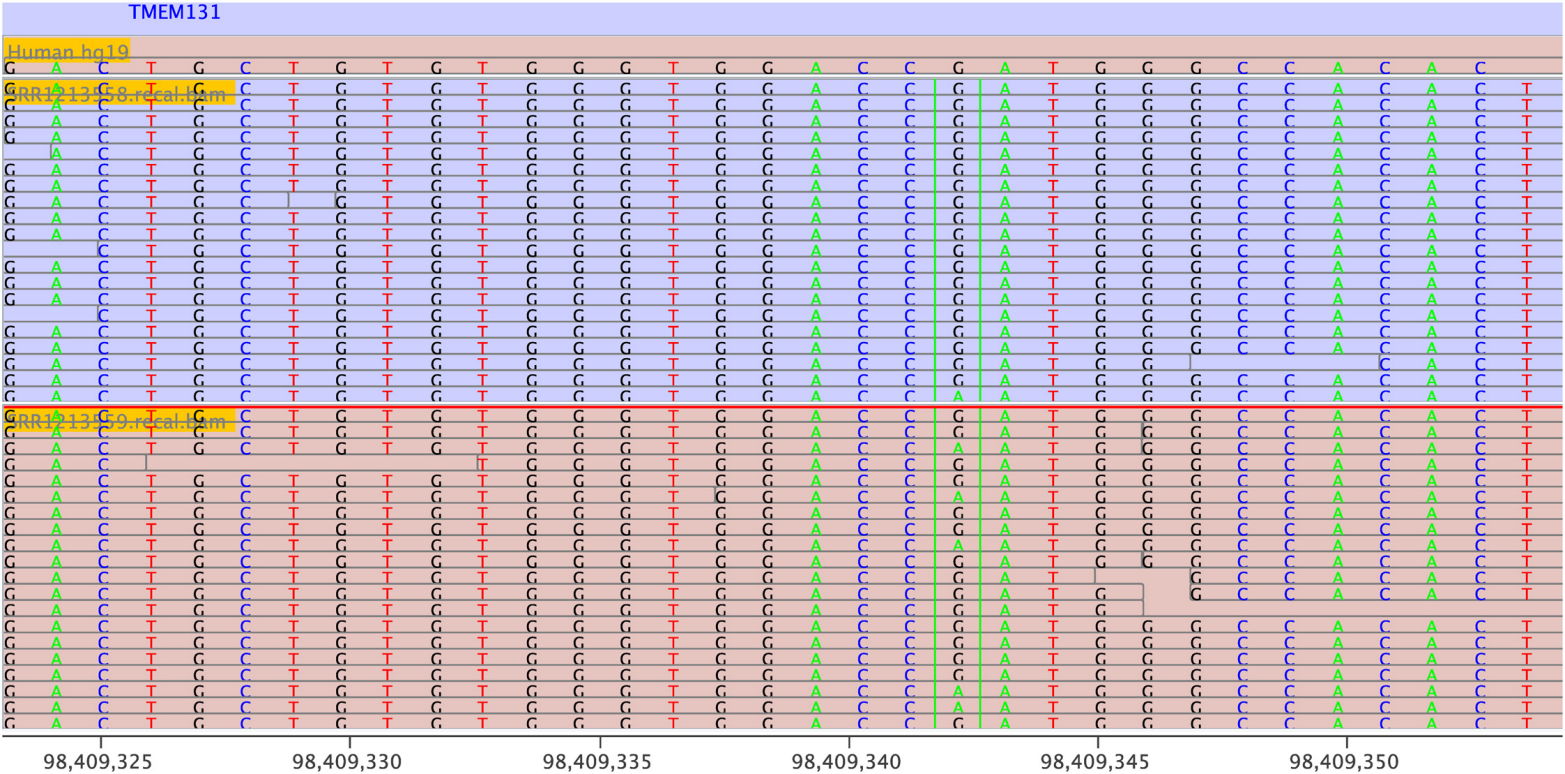
A RNA editing site (chr2:98409343) in *TMEM131* is highlighted in the context of sequenced regions. A G→A change (in the minus strand) was identified by RNA-seq. The upper panel (Run ID: SRR1213558) indicates a normoxia sample and the lower panel is for hypoxia sample (Run ID: SRR1213559).

We first tested 220 C→U (and G→A) sites listed in Supplementary Table S2 in Sharma et al. [6]. Of 220 potential RNA-editing sites, 167 variants sites were called using the GATK guideline. RED in *de novo* mode (MySQL (version 5.1.73), Java (version 1.8.0_25), and R (version 3.0.2)) detected 129 potential RNA-editing sites (*P* < 0.05, Fisher’s exact test). REDItools in *de novo* mode (python version 2.6.6) identified 171 sites (*P* < 0.05). 121 sites (55%) were identified in two programs. Then, we tested the presence/absence of 30 potential RNA-editing sites that affecting amino acid [6]. Of these 30 sites, 28 sites were validated by RT-PCR Sanger sequencing (Table 1 list in Sharma et al. [6]). We counted the number if a given site was identified in any one of the three hypoxic samples. RED in *de novo* mode detected 18 out of 28 true RNA-editing sites (*P* < 0.05, Fisher’s exact test) and one out of two false RNA-editing sites. For comparison, the *de novo* mode in REDItools detected 21 out of 28 true RNA-editing sites (*P* < 0.05) and one out of two false RNA-editing sites. The sensitivity and specificity of the two methods in identifying RNA editing sites are shown in Fig 3, indicating that REDItools had a better sensitivity but similar specificity. The Venn diagram (Fig 3) indicated that 17 sites (60.7%) were identified by both software. A validated RNA-editing site (chr2:98409343 in *TMEM131*) identified by RED is shown in Fig 4.

We noted that different numbers of RNA editing sites were identified between RED and REDItools, partly due to different number variants used in the programs. The sensitivity of variants called by GATK ‘HaplotypeCaller’ (used in RED) and ‘samtools mpileup’ (used in REDItools) differed, as shown in a recent study [28]. Although the procedure of ‘samtools mpileup’ can identify more true positive SNVs, the identified false positive SNVs were approximately ten times higher than GATK ‘HaplotypeCaller’ process. It should be noted that the calling procedure in GATK has taken into account the information about intron-exon split regions.

The comparison of computation costs for REDItools and RED in analyzing six RNA-seq samples [6] was shown in Table 2. In a single thread mode for analyzing these samples, the ‘HaplotypeCaller’ in GATK cost 127 hours to obtain VCF file and RED cost 25.5 hours for identifying RNA editing sites. The time cost of REDitools varied in different samples although eight threads were assigned in computation for each sample.

**Table 2:** Computation cost in REDItools and RED.

Process/sample	REDItools (hrs)^1^	RED (hrs)^2^
HaplotypeCaller	-	127
Data import	-	0.5
SRR1213569	5.3	6.2
SRR1213561	26.2	3.3
SRR1213559	17.3	4.0
SRR1213562	10.5	2.1
SRR1213560	39.9	6.3
SRR1213558	9.9	3.3

^1^, eight threads were assigned;

^2^, single thread.

The time to produce the analysis-ready bam files (i.e., alignment and correction for technical biases) was not included. HaplotypeCaller was used to call variants in a single sample, and the time of 127 hrs is the total time for six samples.

We also identified potential RNA editing sites by comparing the RNA-seq and whole exome sequencing data in the liver normal tissue of a hepatocellular carcinoma patient (http://www.ncbi.nlm.nih.gov/bioproject/273421) [29] by RED. We used the *DNA-RNA* mode with the parameters listed in Table 1. We identified 442 A→G RNA-editing sites and 14% were non-synonymous sites.

## Availability and Future Directions

RED is an integrated GUI for detecting and visualizing potential RNA-editing sites. RED provides multiple rules to filter out spurious RNA-editing sites and visualize the candidate RNA-editing sites. In addition, MySQL made the query of the filtering result efficient, and enabled the storage of RED results for each filtering step, which can be re-analyzed without running filters again. However, RED depends on three computational frameworks of Java Runtime Environment (jre or jdk with 1.6.0_43 or later), MySQL Database Management System (MySQL 5.1.73 or later) and *R* Environment (R 3.0.1 or later). REDItools requires only Python.

The RED package is freely available under the GPLv3 license at the Git repository web-based hosting service (github), https://github.com/REDetector/RED or SourceForge, https://sourceforge.net/projects/redetector.

Future additions to RED will include: 1) information of each site, including coverage, quality, position, and editing level, will be present in the chromosome panel; and 2) functional categories of RNA editing sites will be annotated, and information of chromosome cytoband will be added.

In conclusion, RED is an effective software for the identification and visualization of RNA editing sites from next-generation sequencing data. It is highly flexible, including a variety of filters and stringent statistical assessment, and may provide very reliable sets of RNA editing candidate sites according to the user’s requirements.

## Supporting Information

S1 Text Information on software, database, and command used in the analyses. (DOCX)
