# Supplementary material for: RED: A Java-MySQL Software for Identifying and Visualizing RNA Editing Sites Using Rule-Based and Statistical Filters
Source: PLoS One. 2016 Mar 1;11(3):e0150465. doi: 10.1371/journal.pone.0150465 (PMC4773184; doi:10.1371/journal.pone.0150465)
Supplement: S1 Text — (DOCX) [file pone.0150465.s001.docx]

Supporting Information

The materials include information on the software and the command used in the analysis.

### 1. Software and database information

#### 1.1 Software and its version

- MySQL: version 5.1.73
- Java: version 1.8.0_25
- R: version 3.0.2
- Python: version 2.6.6
- STAR: version 2.4.0e
- GATK: version 3.3.0
- picard: version 1.81
- samtools: version 1.2

#### 1.2 Database and its version

- refGenome_file: genome.fa (eg, UCSC, hg19)
- dbsnp: dbsnp_138.hg19.vcf (NCBI)
- Repeat_file: hg19.fa.out (<http://www.repeatmasker.org>)
- gene annotation file: genes.gtf (<http://genome.ucsc.edu>)
- DARNED database: hg19.txt (<http://darned.ucc.ie>)
- RADAR database: Human_AG_all_hg19_v2.txt (<http://rnaedit.com>)

**We used a sample from Sharma et al. (Run ID: SRR1213560) as an example.**

### 2. Generate the analysis-ready bam file

#### 2.1 Build human genome index

STAR --runMode genomeGenerate --genomeDir <genomeDir> --genomeFastaFiles --runThreadN 40

#### 2.2 Alignment

runDir=SRR1213560.1pass

cd <runDir>

STAR --genomeDir <genomeDir> --readFilesIn SRR1213560_1.fq SRR1213560_2.fq --outFilterMultimapNmax 1 --runThreadN 10

#### 2.3 A new index is created using splice junction information

genomeDir2=SRR1213560.hg19_2pass

STAR --runMode genomeGenerate --genomeDir <genomeDir2> --genomeFastaFiles <refGenome_file> --sjdbFileChrStartEnd <runDir>/SJ.out.tab --sjdbOverhang 75 --runThreadN 10

#### 2.4 The resulting index is used to produce the final alignments

STAR --genomeDir <genomeDir2> --readFilesIn SRR1213560_1.fq SRR1213560_2.fq --runThreadN 10 --outFileNamePrefix SRR1213560.sorted

#### 2.5 Sort sam file and generate the bam file

java -Xmx60g -jar <Picard_dir>/SortSam.jar SO=coordinate INPUT=SRR1213560.sortedAligned.out.sam VALIDATION_STRINGENCY=LENIENT OUTPUT=SRR1213560.sortedAligned.out.bam

samtools index SRR1213560.sortedAligned.out.bam

ln -s SRR1213560.sortedAligned.out.bam SRR1213560.star.raw.bam

samtools index SRR1213560.star.raw.bam

#### 2.6 Add read groups, sort, mark duplicates, and create index with Picard

java -Xmx60g -jar <Picard_dir>/AddOrReplaceReadGroups.jar I=SRR1213560.star.raw.bam O=SRR1213560.rg_added_sorted.bam SO=coordinate RGID=SRR1213560 RGLB=SRR1213560 RGPL=Illumina RGPU=HiSeq RGSM=SRR1213560

samtools index SRR1213560.rg_added_sorted.bam

java -Xmx60g -jar <Picard_dir>/MarkDuplicates.jar INPUT=SRR1213560.rg_added_sorted.bam OUTPUT=SRR1213560.dedupped.bam METRICS_FILE=SRR1213560.metrics REMOVE_DUPLICATES=true ASSUME_SORTED=false TMP_DIR=<TMPDIR> VALIDATION_STRINGENCY=LENIENT

samtools index SRR1213560.dedupped.bam

#### 2.7 Split'N'Trim and reassign mapping qualities

java -Xmx60g -jar <GATK.jar> -T SplitNCigarReads -R <refGenome_file> -I SRR1213560.dedupped.bam -o SRR1213560.split.bam -rf ReassignOneMappingQuality -RMQF 255 -RMQT 60 -U ALLOW_N_CIGAR_READS

samtools index SRR1213560.split.bam

#### 2.8 Determine suspicious intervals which are likely in need of realignment

java -Xmx60g -jar <GATK.jar> -T RealignerTargetCreator -R <refGenome_file> -nt 300 -known <dbsnp> -I SRR1213560.split.bam -o SRR1213560.forRealigner.intervals

#### 2.9 Realigning

java -Djava.io.tmpdir=<TMPDIR> -Xmx60g -jar <GATK.jar> -T IndelRealigner -R <refGenome_file> -known <dbsnp> -targetIntervals SRR1213560.forRealigner.intervals -I SRR1213560.split.bam -o SRR1213560.realn.bam

samtools index SRR1213560.realn.bam

#### 2.10 Base quality score recalibration

java -Xmx60g -jar <GATK.jar> -T BaseRecalibrator -R <refGenome_file> -knownSites <dbsnp> -I SRR1213560.realn.bam -cov ReadGroupCovariate -cov QualityScoreCovariate -cov CycleCovariate -cov ContextCovariate -o SRR1213560.recal_data.grp

#### 2.11 PrintReads

java -Xmx60g -jar <GATK.jar> -T PrintReads -R <refGenome_file> -I SRR1213560.realn.bam -BQSR SRR1213560.recal_data.grp -o SRR1213560.recal.bam

samtools index SRR1213560.recal.bam

### 3. Generate the analysis-ready VCF file

#### 3.1 Variants calling (SNPs and INDELs) for a single sample

java -Xmx30g -jar <GATK.jar> -T HaplotypeCaller -R <refGenome_file> -I SRR1213560.recal.bam -dontUseSoftClippedBases -stand_call_conf 20.0 -stand_emit_conf 20.0 -o SRR1213560.raw.vcf --output_mode EMIT_ALL_SITES;

#### 3.2 Apply hard filters to the RNA snvs/indels (optional)

java -Xmx30g -jar <GATK.jar> -T VariantFiltration -R <refGenome_file> -V raw.snps.indels.vcf -window 35 -cluster 3 -filterName FS -filter "FS > 30.0" -filterName QD -filter "QD < 2.0" -o SRR1213560.raw.hardfiltered.vcf

The files of SRR1213560.recal.bam (as well as other analysis ready bam files) and SRR1213560.raw.hardfiltered.vcf were used in RNA-editing sites detection

### 4. RED procedure

#### 4.1 Run RED with GUI

java -Xmx8g –jar RED.jar

#### 4.2 Run RED in command line

java -jar <RED.jar> -H localhost -p 3306 -u username -P {PWD} -d DENOVO_SRR1213560 -m denovo -t all -o ./denovo_SRR1213560 -r /usr/bin/Rscript -E chrom,pos,strand,ref,alt,alu -O 513426 --rnavcf=SRR1213560.raw.hardfiltered.vcf --repeat=<repeat_file> --splice=<gene annotation file> --dbsnp=<dbsnp> --darned=<darned database> --radar=<radar database>

### 5. REDItool procedure

python <REDItoolDenovo.py> -i SRR1213560.recal.bam -f <refGenome_file> -t 8 -o reditools_out -a t -c 6 -Q 33 -V 0.05 -w BH
